# The outcome of polyethylene glycol fusion augmented by electrical stimulation in a delayed setting of nerve repair following neurotmesis in a rat model

**DOI:** 10.1007/s00701-023-05854-6

**Published:** 2023-11-01

**Authors:** Nanda Acharya, A. M. Acharya, Anil K. Bhat, Dinesh Upadhya, Dhiren Punja, Sumalatha Suhani

**Affiliations:** 1 Department of Physiology, Kasturba Medical College, Manipal Academy of Higher Education, Manipal, Karnataka India 576104; 2 Department of Hand Surgery, Kasturba Medical College, Manipal Academy of Higher Education, Manipal, Karnataka India 576104; 3 Centre for Molecular Neurosciences, Kasturba Medical College, Manipal Academy of Higher Education, Manipal, Karnataka India 576104; 4 Department of Anatomy, Kasturba Medical College, Manipal Academy of Higher Education, Manipal, Karnataka India 576104

**Keywords:** Neurotmesis, Wallerian degeneration, Nerve regeneration, Polyethylene glycol, Electrical stimulation

## Abstract

**Purpose:**

Polyethylene glycol is known to improve recovery following its use in repair of acute peripheral nerve injury. The duration till which PEG works remains a subject of intense research. We studied the effect of PEG with augmentation of 20Htz of electrical stimulation (ES) following neurorrhaphy at 48 h in a rodent sciatic nerve neurotmesis model.

**Method:**

Twenty-four Sprague Dawley rats were divided into 4 groups. In group I, the sciatic nerve was transected and repaired immediately. In group II, PEG fusion was done additionally after acute repair. In group III, repair and PEG fusion were done at 48 h. In group IV, ES of 20Htz at 2 mA for 1 h was added to the steps followed for group III. Weekly assessment of sciatic functional index (SFI), pinprick, and cold allodynia tests were done at 3 weeks and euthanized. Sciatic nerve axonal count and muscle weight were done.

**Results:**

Groups II, III, and IV showed significantly better recovery of SFI (II: 70.10 ± 1.24/III: 84.00 ± 2.59/IV: 74.40 ± 1.71 vs I: 90.00 ± 1.38) (*p* < 0.001) and axonal counts (II: 4040 ± 270/III: 2121 ± 450/IV:2380 ± 158 vs I: 1024 ± 094) (*p* < 0.001) at 3 weeks. The experimental groups showed earlier recovery of sensation in comparison to the controls as demonstrated by pinprick and cold allodynia tests and improved muscle weights. Addition of electrical stimulation helped in better score with SFI (III: 84.00 ± 2.59 vs IV: 74.40 ± 1.71) (*p* < 0.001) and muscle weight (plantar flexors) (III: 0.49 ± 0.02 vs IV: 0.55 ± 0.01) (*p* < 0.001) in delayed repair and PEG fusions.

**Conclusion:**

This study shows that PEG fusion of peripheral nerve repair in augmentation with ES results in better outcomes, and this benefit can be demonstrated up to a window period of 48 h after injury.

## Introduction

Peripheral nerve injuries are a significant problem affecting approximately a million patients requiring surgery worldwide every year [8]. The recovery is mainly influenced by factors like severity, extent and type of injury, time from injury to treatment, and age of the patient [15]. The challenge in these injuries is Wallerian degeneration following which axons regenerate at an abysmally slow rate of only 1 mm/day following complete nerve division or neurotmesis [7, 8, 16].

Recent literature has shown immense potential in two methods showing better functional recovery after peripheral nerve injury repairs in animal models. One of them is using polyethylene glycol (PEG) at the site of nerve repair in an acute setting as a fusogen [7]. Its use has been shown to result in the fusion of the divided axons resulting in immediate restoration of the axonal continuity and subsequent near-complete recovery of function within 6 weeks in rodent models [4, 7]. An essential criterion for this fusion is that Wallerian degeneration should not have set in, and hence, this is effective only in the immediate few hours after injury [4, 7]. However, in clinical scenarios, the nerve injury is compounded by the presence of polytrauma, contaminated injuries, doubtful or missed diagnosis, and delayed presentation where late surgical repair is often inevitable where Wallerian degeneration would have set in [8, 16]. Experimentally, PEG is shown to be an effective fusogen when used within 24 h in rat models following nerve injury [4]. The maximum time beyond which fusion may not happen is still a research query [7]. This drug is also an effective sealant at nerve repair sites encouraging a better environment for axonal sprouting and transport across the distal nerve segment in case fusion does not happen, particularly in relatively delayed cases [7].

The other method is the intra-operative use of electrical stimulation (ES) of nerves after repair [2, 10, 12, 21]. Experiments in animal models and more recently clinical trials in humans have shown the effectiveness of 20 Hz electrical current stimulation at the proximal end of the nerve repair site for 1 h [2, 10, 12, 21]. This has been shown to encourage a dramatic and selective increase in the volume of motor axonal sprouts entering the distal end of the repaired nerve, resulting in a superior quality of recovery of motor function [2, 10, 12, 21]. The ES has shown to be effective for sensory axonal recovery and in a delayed setting as well [10, 12].

To mimic the clinical scenario of delayed nerve repair, we hypothesized that there may still be some viable axon at the distal segment after neurotmesis, which can be fused with PEG at 48 h. The remaining axons which have started undergoing the process of Wallerian degeneration can be encouraged to achieve an enhanced quality of recovery with ES, which is well known to induce the generation of a larger volume of axonal sprouts at the site of repair. Hence, our research question was whether PEG fusion of peripheral nerve works better with augmentation of electrical stimulation in a delayed setting of 48 h after nerve injury to achieve improved results compared to the traditional repair of nerves. To test this hypothesis, we studied the comparative functional recovery of PEG fused sciatic nerve repair at 48 h after injury with an additional intervention of per-operative ES of nerve proximal to the site of repair at 20Htz for 1 h with a control group of traditional repair and with immediate fusion with PEG and delayed fusion with PEG. The aim was as follows: (1) to evaluate the effectiveness of the PEG fusion of peripheral nerve neurotmesis repaired at 48 h and (2) the augmentative effect of ES on the recovery after delayed repair and PEG fusion at 48 h. Presumably, in such a scenario, ES will improve the volume of axons in the distal segment in a PEG-sealed nerve repair.

## Methods

The study was performed at the university animal house facility after approval of the institutional animal ethics board following the ‘ARRIVE’ guidelines. It was performed in compliance with the standard of ethics mentioned in the Declaration of Helsinki (1964) and its amendments. We selected 24 adult female Sprague Dawley rats (weight, 200–250 gms) for our study. A total of four groups were formed with six rats in each group.

The sample size was determined based on previous studies describing individual groups of six each at a minimum of two time points, providing adequate power to detect differences of ≥ 20% with *p* ≤ 0.05 between the control and experimental groups [15].

The rats were trained for walking track assessment for 1 week before surgery and assessed for the sciatic functional test (SFI) based on the technique suggested by Bain et al. [3]. Baseline footprints for SFI were documented on the previous day of surgery [15]. Three consecutive footprints were documented from which measurements of toe spread (TS), intermediary toe spread (IT), and print length (PL) were measured on the experimental (E) and unoperated (N) limbs [3]. The SFI was then calculated using the formula: SFI =  − 38.3([EPL − NPL]/NPL) + 109.5 ([ETS − NTS]/NTS) + 13.3 ([EIT − NIT]/NIT) − 8.8 [3]. An SFI score for the normal nerve approaches or equals the value of zero, whereas − 100 is suggestive of the total loss of sciatic nerve function.

### Procedure

The animals were given a gavage of non-steroidal anti-inflammatory drug, carprofen (5 mg/kg /OD), 1 h before surgery. They were divided into four groups, each consisting of six animals. Group I represented the controls undergoing routine nerve transection and traditional epineural repair under the microscope immediately. Group II underwent nerve transection, repair, and PEG fusion immediately. Group III underwent nerve transection, but repair and PEG fusion were done after 48 h. Group IV underwent the same as group III but was additionally subjected to per-operative electrical stimulation of 20 Htz for 1 h proximal to the site of nerve fusion.

**Fig. 1:**
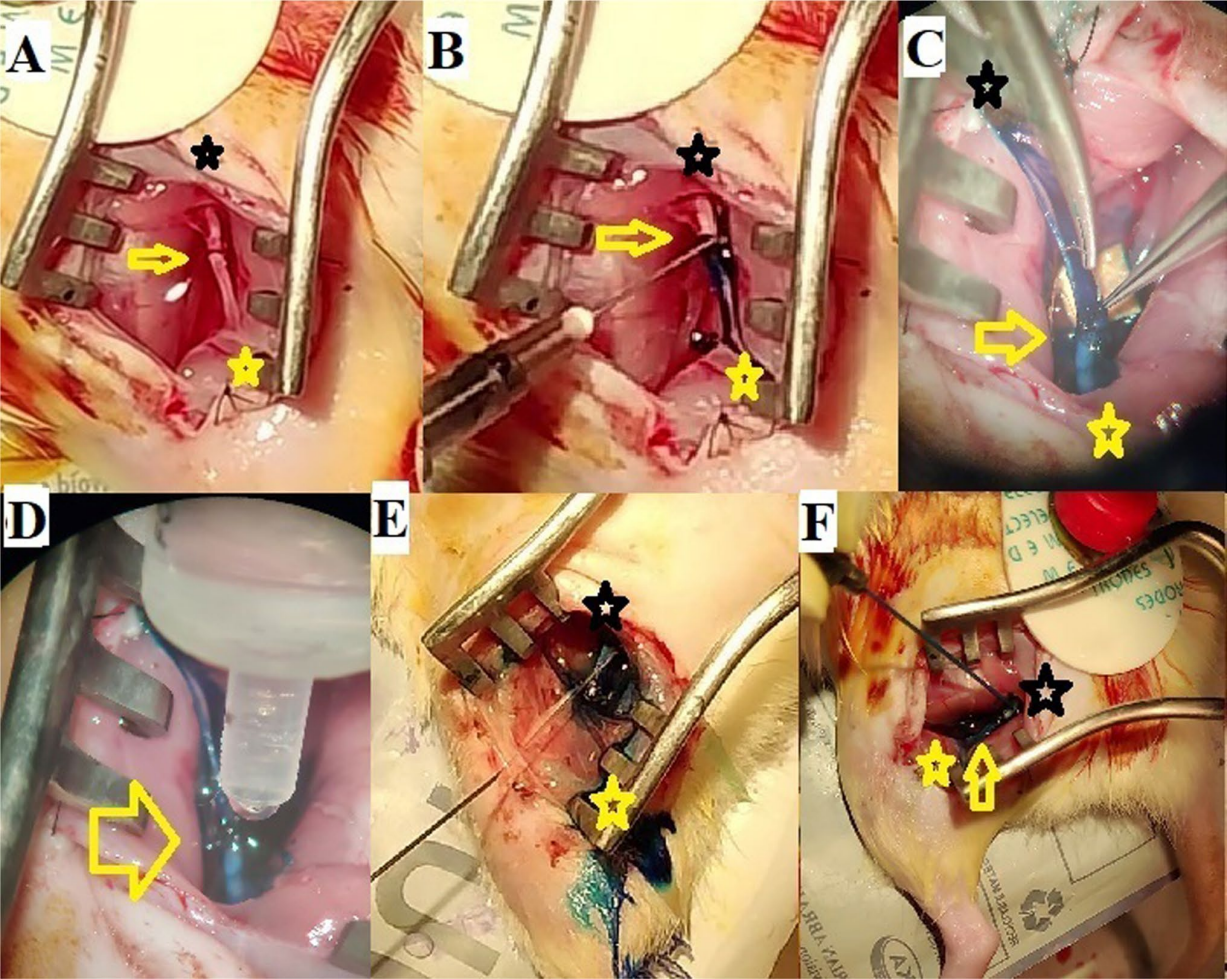
In all the images, note that the proximal end is represented by the black star, the distal end by the yellow star, and the site of neurotmesis and repair by the yellow arrow. **A** Shows the application of the hypotonic calcium solution Plasmalyte-A. The site of neurotmesis of the sciatic nerve is shown in the yellow arrow. **B** Shows the application of Methylene Blue at the site of cut ends of the nerve. **C** Shows the application of 10–0 nylon epineural sutures to repair the nerve under the microscope. **D** Shows the application of 50% PEG solution on the site of suture repair. **E** Shows the application of Ringer lactate solution to flush out the PEG and seal the repair site. **F** Shows the needle giving 1 h of 20 Htz ES to the proximal end of the repair site of PEG fused nerve repair

The animals were anesthetized with an intraperitoneal injection of a cocktail containing xylazine (10 mg/kg) and ketamine (75 mg/kg). Under aseptic conditions, the sciatic nerve was exposed. In groups I and II, the sciatic nerve was transected midway between the sciatic notch and its distal trifurcation. A primary epineural neurorrhaphy with two sutures was done under a microscope with 10–0 nylon sutures (day 0) in group I (controls). In group II, PEG fusion was done as follows: The nerve endings, after transection, were irrigated with a hypotonic solution, Plasmalyte-A™ (Baxter) (Fig. 1A) [7, 11]. This removes the Ca^2+^ ions which are known to seal and close the axon ends immediately after the nerve is cut by forming intracellular vesicles [7, 11]. The hypotonic saline would expand the open axonal ends, which could then be prepared for apposition by PEG-fusion to the distal cut end [6, 9]. This is immediately followed by the application of 1% methylene blue in sterile water at both nerve ends, which is an antioxidant and maintains the Ca^2+^ free environment and prevents additional vesicle formation (Fig. 1B) [7, 11]. Primary neurorrhaphy is then done by the same technique as for group I. It provides mechanical strength at the repair site and prevents PEG-fused axons from pulling apart as the plasmalemma has a low tensile strength (Fig. 1C) [7, 11]. We then applied a solution of 50% PEG (Sigma-Aldrich, 3.35 kD) to the site of repair for a minute (Fig. 1D) [6, 10]. The wound was rinsed with the calcium-containing Ringer’s lactate solution, to remove the residual PEG solution and provide Ca^2+^ to repair axolemmal holes with vesicles (Fig. 1E) [7, 11]. In groups III and IV, the nerve was crush ligated with 6–0 nylon sutures to achieve neurotmesis at the same site as described for groups I and II. After 48 h, the ligature was reexplored and divided at the ligation site and the segment was examined under the microscope to confirm damage to all the fascicles. The ends were freshened with microscissors followed by neurorrhaphy and PEG fusion in the same way as done for group II. In group IV, we additionally gave ES of 20Htz at 2 mA using a manual electrical stimulator (Indmed™) for 1 h immediately following the repair at a site 5 mm proximal to the repair site (Fig. 1F) [2, 9, 11, 20]. The wound was closed with 4–0 nylon sutures followed by subcutaneous infiltration of gentamycin (2 mg/kg.). The animals were returned to the housing facility and observed for complications like wound dehiscence and chest or gastrointestinal infections. Carprofen was given orally as an analgesic for 5 days.

### Pinprick test

**Fig. 2:**
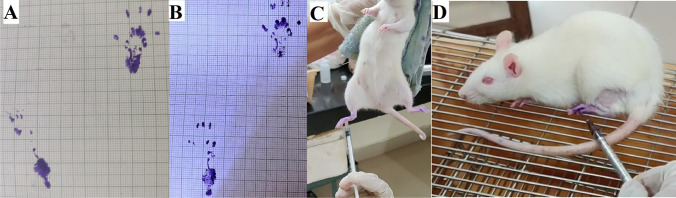
SFI was assessed on the seventh, 14th, and 21st days (Fig. 2A and B). The pinprick test and cold allodynia test were evaluated on the 21st day (Fig. 2C and D). **A** Shows the hind limb print of the rat for SFI, which underwent PEG fusion and ES of the sciatic nerve after neurotmesis in the first week. Note the footprint of the left foot, which is paralyzed. The right side is normal. **B** Shows the foot position improvement, increased toe spread length (TS), and decreased plantar length (PL) at 3 weeks. **C** Showing application of a drop of acetone in cold allodynia test. **D** Showing the method of the pinprick test

The pinprick test was done to assess the sensory recovery in the feet (Fig. 2D). The unoperated foot was tested with a 25-G needle pinprick stimulus to document the normal reaction, represented by an immediate foot withdrawal, and compared with the operated side. The foot was pricked on the plantar side of the lateral half. The responses were graded as suggested by Paskal et al. [17] ( −) no response; ( +) mild response, a very weak retraction or trembling of the leg; (+ +) moderate response; and (+ + +) normal response, as observed on the healthy side [17]

### Cold allodynia test

At 3 weeks post-operatively, animals were tested for cold allodynia response, as suggested by Jo et al. (Fig. 2C) [13]. The animal was placed on a meshed metal grid for 5 min and allowed to familiarize itself with the setup. A drop of acetone was placed using a syringe without its needle on the plantar side of the operated foot. The animal’s reaction was observed for a minute [14]. Generally, there is no response or a minimal reaction to cold acetone applied to the foot pad of normal rats [14]. Similarly, no response will be seen with absent sensations. However, after nerve injury, typical behavioral responses can be observed with partial recovery on applying cold acetone, indicating increased cold sensitivity. A recovering or partially recovered nerve will show paw-licking/shaking behavior. This is accepted as a positive response to acetone suggesting sensory recovery [14]. The total time of behavior exhibited by the animal was also measured.

On the 21st day, all the animals were euthanized, and the sciatic nerve samples of a length of 1 cm immediately distal to the site of repair were collected for hematoxylin and eosin-based histological study for axonal counts. The legs’ gastrosoleus and extensor compartment muscles (tibialis anterior and extensor digitorum longus and hallucis longus) were checked for their weights using a digital weighing scale. The affected to contralateral side muscle weight ratio was documented and recorded as relative muscle mass.

### Nerve axonal count

The sciatic nerve sample was fixed in 4% paraformaldehyde in phosphate-buffered saline (PBS) for 3 h [9]. Before embedding, the nerve sample was washed and preserved in 0.2 g glycine in 100 mL PBS. It was dehydrated serially with 50, 70, 80, 95, and 100% ethanol passages and embedded in paraffin [9]. A 7- to 8-mm thick series of transverse sections were cut using a Leica™ microtome, placed on slides, and put for overnight drying. They were then deparaffinated and rehydrated with ethanol in decreasing strength [9]. The slide was immersed in 0.1% hematoxylin for 10 min and washed in water for 15 min. It was then washed in distilled water after immersion in 0.1% eosin for 5 min. The slides were then analyzed at 10 × and 50 × on an Olympus microscope. The open-source ImageJ (NIH, Bethesda) software was used for nerve fiber counts. The muscle sample was sent for weight measurement analysis using a weighing scale. The analysis was done by an observer blinded to the four groups.

### Statistical analysis

All statistical analyses were performed by Jamovi software. Data was entered as mean ± SD. Data for SFI was assessed with a two-way analysis of variance (ANOVA). The differences between groups in the axonal count and the functional test results were verified with an unpaired *T*-test. The threshold of statistical significance was set at *p* ≤ 0.05.

## Results

### Sciatic functional index

As per Table 1, the pre-operative SFI was uniformly normal and equally distributed in all four groups. One week after the surgery on the affected side, high values of SFI confirm the neurotmesis in all the groups, suggesting a lack of motor recovery across all the groups. The second-week values show a recovery pattern that is significantly better (*p* < 0.001) in all the intervention groups (Table 1) when compared with the control. In the third week, all three intervention groups showed significantly better recovery (*p* < 0.001) in comparison to control (Table 1). The results showed a better recovery pattern for the experimental groups when compared to the control (Group I) (Fig. 2A and B). This suggests that a good volume of axons may be available for PEG fusion at 48 h after neurotmesis. Group II showed the most improved results, followed closely by group IV. Tukey post hoc tests at 3rd week between groups III and IV suggest a significantly better motor recovery (*p* < 0.001) in group IV. We observed that using electrical stimulation helped improve results in the delayed repair at 48 h.

**Table 1:** Table showing the values for SFI for the four groups

Period	SFI score ± SD				*p*
	0th hour control	0th hour PEG^##^	48 h PEG	48 h PEG + ES^#^	
Pre-Op	8.80 ± 3.69	10.55 ± 4.31	8.33 ± 5.15	11.10 ± 5.58	0.702
Week_1	84.10 ± 2.72	80.20 ± 1.15	82.80 ± 2.46	81.40 ± 1.29	0.019
Week_2	87.70 ± 1.66	76.00 ± .80	84.30 ± 2.41	80.20 ± 1.18	< .001*
Week_3	90.00 ± 1.38	70.10 ± 1.24	84.00 ± 2.59	74.40 ± 1.71	< .001*

^*^Expressed values are significant at *p* < 0.05. ^#^*ES* electrical stimulation, ^##^*PEG* polyethylene glycol

### Skin pinprick test

**Fig. 3:**
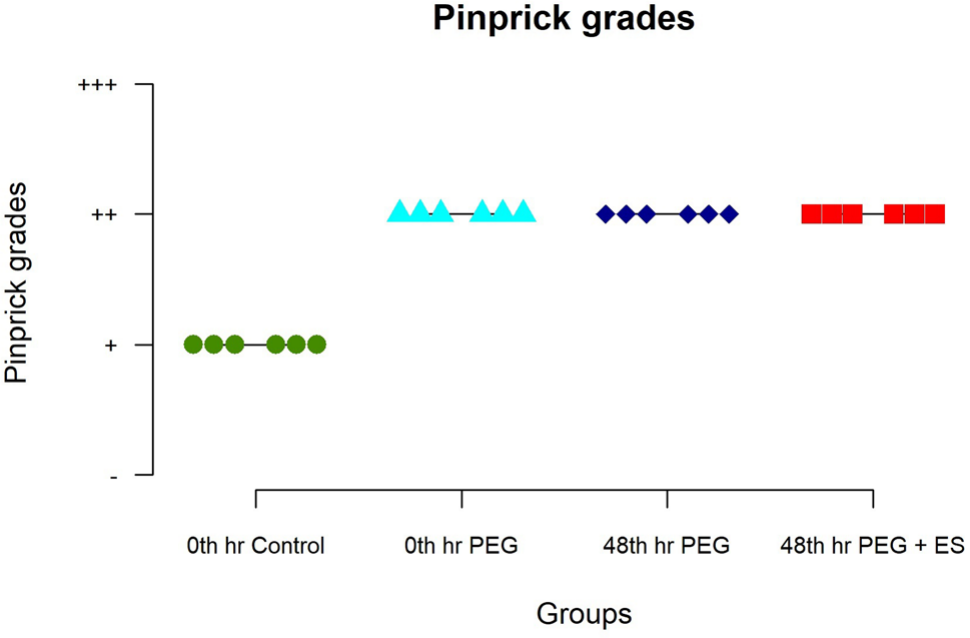
Showing the plot diagram of the results of pinprick at 3 weeks. Note the better response of the experimental group when compared to the controls

The skin prick test result was considered a measure of the sensory function return. One week after the surgery, none of the study animals responded to the foot prick stimulus. Pinprick grades were higher in the experimental groups than in the control group, but the difference was not statistically significant among the experimental groups (*p* = 0.01) (Table 2) (Fig. 3). The results suggest that partial recovery of sensation does occur even by 3 weeks which shows an overall higher value in the experimental groups. The recovery in groups III and IV is as good as in the acute PEG repair group (*p* = 0.01).

**Table 2:** Table showing the values for parameters tested for the four groups

Group	Pinprick	Cold allodynia (seconds)	Dorsi flexors (relative muscle mass ratio)	Gastrosoleus (relative muscle mass ratio)	^#^Axon count/ 250 µm^2^	
0th_hr_Control	1 ± 0	00 ± 0.0	0.34 ± 0.02	0.37 ± 0.01	1024 ± 094	*p* < .001*
0th_hr_PEG	2 ± 0	35 ± 1.7	0.48 ± 0.06	0.54 ± 0.02	4040 ± 270	
48th_hr_PEG	2 ± 0	45 ± 1.8	0.45 ± 0.02	0.49 ± 0.02	2121 ± 450	
48th_hr_PEG_plus_ES	2 ± 0	39 ± 1.8	0.48 ± 0.01	0.55 ± 0.01	2380 ± 158	

^*^Expressed values are significant at *p* < 0.05. ^#^Reference count in the normal sciatic nerve was 5540/250 µm^2^

### Cold allodynia

**Fig. 4:**
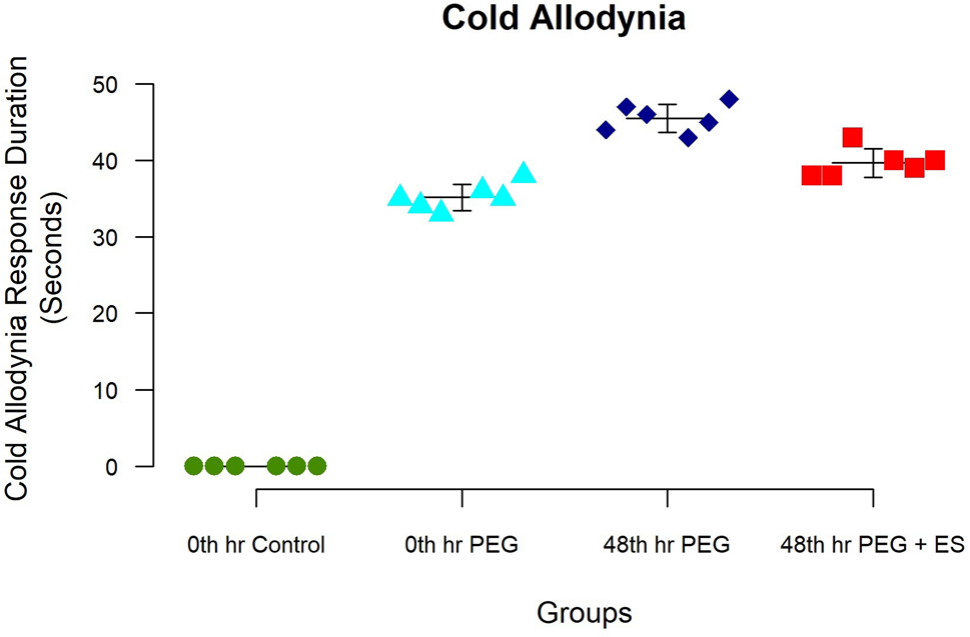
The plot diagram shows the response to cold allodynia using acetone at 3 weeks. The experimental group can observe sensory response as early as 3 weeks

Groups II, III, and IV showed a response to cold stimuli. The cold allodynia response was absent in the control group, suggesting that sensation recovery had not happened by 3 weeks. In the unpaired *T*-test, a comparison of groups III and IV demonstrates that a significant early response was seen in group IV (*p* < 0.001) (Fig. 4). The intervention groups showed a trend toward faster recovery of sensation in comparison to the control group. The difference in the cold allodynia response was insignificant between groups II and IV (*p* = 0.01), suggesting a similar response of sensory recovery. This shows the possible benefit of adding ES to the regime of PEG fusion in delayed repairs.

### Relative muscle mass

**Fig. 5:**
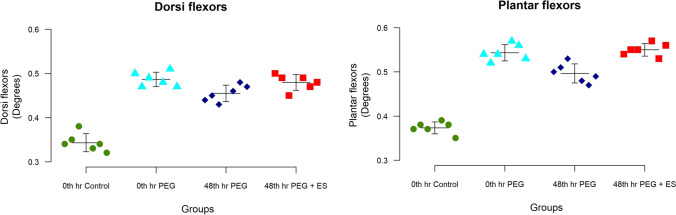
Plot diagram showing the relative muscle mass in the gastrosoleus and dorsiflexor groups. Notice the higher values of mass for the experimental group

The relative muscle mass ratio for both the gastrocnemius(plantar flexors) and ankle and toe extensor muscles (dorsiflexors) remained lower than the contralateral normal side, suggesting that Wallerian degeneration cannot be prevented entirely even after acute PEG fusion (Table 2). However, the value of all the experimental group animals was more than the control. In the unpaired *T*-test, a comparison of groups III and IV demonstrates a significantly higher weight of plantar flexors in group IV (*p* < 0.001) (Fig. 5). Similarly, in the unpaired *T*-test for dorsiflexors, a comparison of groups III and IV demonstrates a higher weight of dorsiflexors in group IV (*p* < 0.05) (Fig. 4). The difference was better reflected for the plantar flexors among the intervention group when compared to the control. An important reason which could explain this is that the nerve to the gastrosoleus is closer to the site of repair and may have recovered faster to show a marginal improvement in its volume.

### Nerve axonal count

**Fig. 6:**
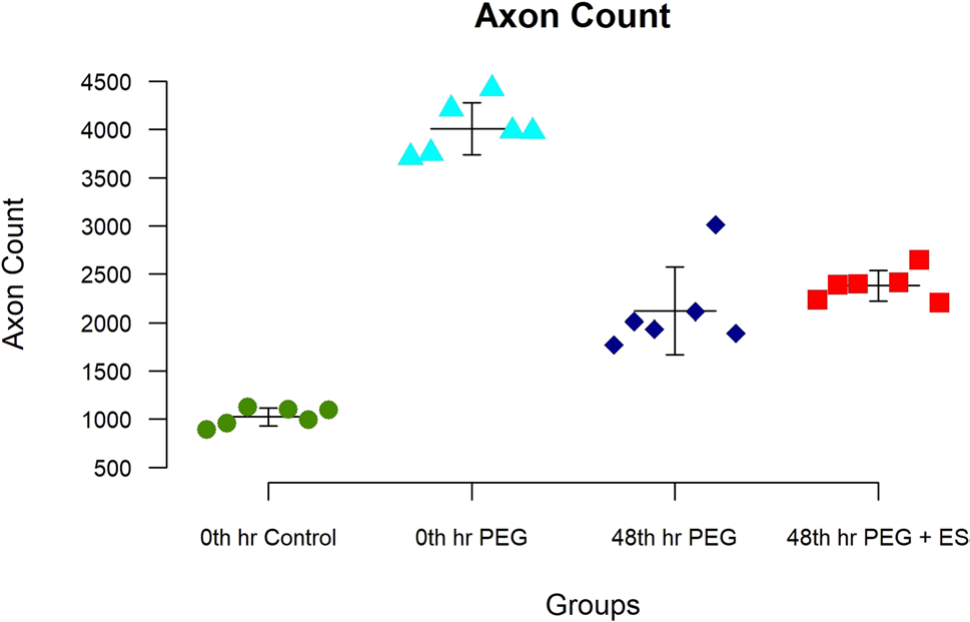
Showing the axonal count at 3 weeks. Note that this count is better for the experimental group. Note that the delayed group also shows a better improvement

**Fig. 7:**
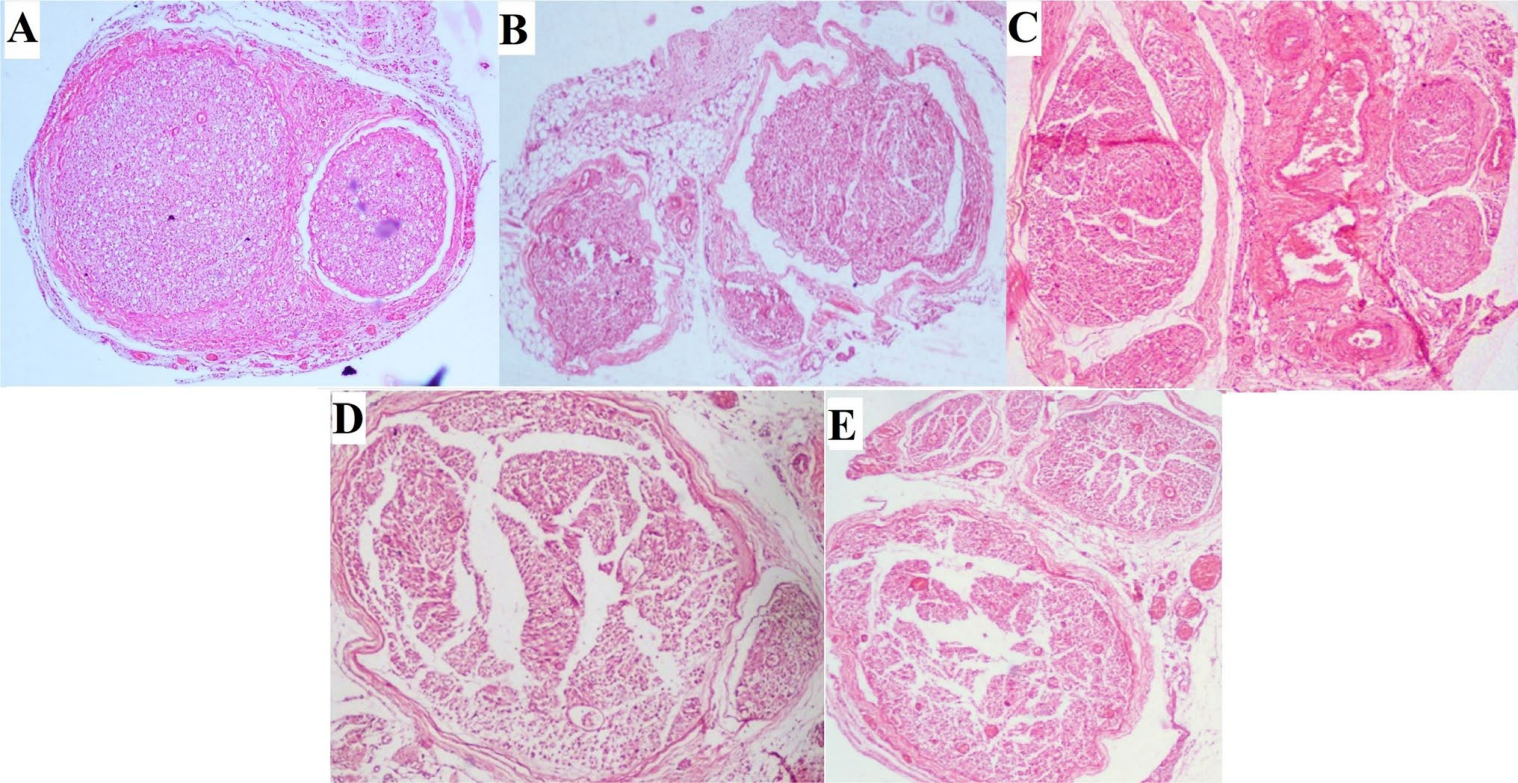
**A** Showing the histological section of the normal anatomy of the sciatic nerve (10 ×). Notice the volume of axons present in the two oligofascicles. The axons are shown as dots in the space’s center, representing the lipid-rich myelin. **B** Notice the collapsed and degenerated segment of the denervated nerve (control). The axons are replaced by surrounding mesodermal tissue. **C** Notice the distal segment section of the acute PEG fused nerve. There is a significant volume of axons that have not undergone Wallerian degeneration. **D** and **E** Show the section of 48th hour PEG fusion (**D**) and augmented with ES (**E**). This section shows the presence of a good volume of axons

In this series, total nerve fiber counts (the number of axons) increased distal to the repair site in all three groups treated with PEG compared with the repair-alone control group (Table 2). One-way ANOVA results showed a significant difference between the control (group I) and the other groups for the axon count during 3rd week (*F* = 117.82, *p* < 0.001) (Table 1) (Figs. 6 and 7). In the unpaired *T*-Test comparison of groups III and IV, there is a mild increase in axon count in group IV, and no statistical significance is seen.

When the efficacy of interventions applied for delayed settings is compared between groups III and IV, group IV demonstrates significant therapeutic efficacy, as seen for SFI, dorsiflexors, and plantar flexors. Early response is seen for cold allodynia tests underlining the benefits of ES.

## Discussion

We hypothesized that there may still be some axons that can be viably fused at 48 h. This may extend the nerve repair window period, allowing for preliminary medical stabilization of patients with additional trauma. Our study reconfirmed improved results for functional recovery and histological counts in the acute PEG fusion group. The results also showed better functional recovery, even for the delayed repair groups, compared to the control. Successful PEG fusion at 48 h has not been reported earlier in literature, and this technique would help in clinical applications in humans for cases with delayed presentation or missed diagnosis of neurotmesis. The improved motor function was shown by SFI, sensory function assessed with pinprick and cold allodynia tests and axonal count assessment were comparable for acute and delayed repair groups. Results demonstrated the feasibility of viable restoration of axonal continuity even at 48 h and also the beneficial role of electrical stimulation.

The fundamental problem after neurotmesis is the delayed recovery after nerve repair due to Wallerian degeneration of the distal segment and slow progression of the axons across the endoneurial tubes. In 2012, Bittner et al. successfully used a PEG-fusion protocol in vivo to demonstrate morphological continuity after nerve repair [6, 7]. They showed this dramatic ability to restore immediate continuity in PEG-fused axons with evidence from intra-axonal dye diffusion and electrical continuity shown by recorded compound action potentials (CAPs) across the repair site. Since then, there have been numerous reports of successful demonstrations in animal models and humans on restoring immediate axonal continuity following PEG fusion of repaired nerves which bypasses the concept of inherent Wallerian degeneration and prolonged healing time [4–7, 11, 14, 17–19]. This has resulted in markedly improved results with a faster recovery rate following nerve repair, as demonstrated in animal models.

However, for a successful fusion, the repair should be accomplished before Wallerian degeneration sets in [7, 11]. There is still a lack of information on how long it takes for complete Wallerian degeneration to set in with reports suggesting a time of 24–72 h [7]. This fact suggests that PEG fusion of injured nerves may be feasible for at least 3 days. Bamba et al. have shown that CAP conductivity can be restored across the repair site up to a day after injury [4]. In an experiment that involved sciatic nerve repair in Sprague Dawley rats at one, eight, and a delayed stage of 24 h, they demonstrated that SFI scores and axonal counts were higher for PEG-treated rats than controls. The results of PEG fusion at 24 h were as impressive as at 1 h. There are reports that this interval might further be extended to 10 days by in vitro cooling of axons or in vivo preservation of nerve in a body part or treatment with cyclosporin A [6, 7]. However, such maneuvers may not be practically feasible.

Literature reports show vast improvement with PEG fusion in sensorimotor function, proven by behavioral and histomorphometric studies. However, these results do not reach pre-operative normal levels, and the overall axonal counts are lower in comparison [7, 19]. An important reason for this has been attributed to a significant volume of axons that do not fuse and undergo PEG sealing and Wallerian degeneration [6, 7, 18, 19]. In addition, the fusion is always indiscriminate between individual axons and does not happen based on corresponding proximal and distal fascicles of specific motor/sensory axons [6, 18, 19]. This information could explain the incomplete sensorimotor recovery we observed in our experimental groups.

In this regard, the role of ES in peripheral nerves becomes important. In 2000, al Majed et al. reported a unique study on the efficacy of ES in promoting and accelerating axonal regeneration and reinnervation through selective motor pathways [1, 2]. They observed a prolonged period of axonal outgrowth in standard animal model nerve repairs that contributes to delayed regeneration of axons over 10 weeks which they referred to as staggered regeneration. Subsequently, after a few weeks of delay, preferential motor reinnervation (PMR) happens where motor axons specifically enter selective motor pathways bypassing sensory pathways. They demonstrated a dramatic reduction of staggered regeneration to 3 weeks with 20 Hz continuous ES for an hour on parent axons proximal to the repair site and accelerated PMR [22]. This was due to changes guided by the nerve’s cell body [20]. Since then, numerous animal experiments have shown ES’s benefits, including improved behavioral and histomorphometry parameters reflecting enhanced functional recovery [2, 10, 12, 21]. Subsequently, this principle was tested in humans in a randomized control trial in the carpal tunnel release procedure [12]. A stimulating electrode wire was placed next to the median nerve proximal to the decompression site, and a 20 Htz ES was given in the immediate post-operative period for 1 h. At the end of 1 year, the motor and sensory functions significantly improved, showing proof of concept [12].

We wanted to observe whether ES augments the benefits of delayed PEG fusion. A significant improvement was seen in the experimental groups’ behavioral and histological axonal counts compared to controls. The sensory recovery, as observed with pinprick and cold allodynia tests and relative muscle mass, also showed a trend toward improved values. The observation of improved function without increased muscle mass is similar to those observed in previous reports [14]. The recovery in the delayed group was much better than control and similar to that of acute PEG repair, which suggests that this technique may be feasible for clinical application in delayed nerve repair settings, although this will need further tests for confirmation.

The limitation of this study is that the results of nerve repair are more dependent on behavioral assessment. Numerous reports have raised questions on the reliability of SFI and cold allodynia tests [6, 14, 18]. However, the behavioral tests remain one of the most accepted and quoted parameters for assessing functional recovery [4–7, 11, 14–16]. Most current reports have assessed the functional recovery from 6 weeks to 6 months [10–18]. However, we decided to do the final assessment of the functional recovery at 3 weeks. In earlier reports, changes of significance were noticed at 3 weeks with respect to nerve repair with both PEG-fusion and ES. Accordingly, our results justify the time chosen for evaluation. In a case of cut injury of the nerve, we would have expectedly observed the cascade of Wallerian degeneration to begin immediately. In our series, the initial injury of nerve was a crush ligation with a suture. The purpose of this technique was to maintain length and ease of repair at 48 h. However, we did not follow the standard concept of simple crush with forceps, which would be an axonotmesis model. We checked this at 48 h also clinically before the second surgery. The paralysis was complete. Per-operatively, the crush ligation site was complete and no fascicles were found intact during resection and freshening of ends under the microscope. Although we took all the precautions and confirmation to ensure an adequate neurotmesis, there remains a theoretical possibility of physical continuity of some of the axons.

The current study establishes the role of PEG fusion and electrical stimulation in a delayed setting of 48 h open new areas for augmentative protocols enhancing nerve regeneration. A more elaborate study design with a longer follow-up would provide further evidence of its benefits and answer questions on the mechanism of action of ES on PEG-fused nerves. A detailed assessment of sensorimotor recovery would require a longer follow-up, which would support an immunohistochemical evaluation of the recovery pattern for sensory and motor axons.

In conclusion, our study shows that PEG fusion of peripheral nerve repair in augmentation with ES results in better outcomes, and this benefit can be demonstrated up to a window period of 48 h after injury.

## Data Availability

Data will be available on request.
